# Condition‐dependent male copulatory courtship and its benefits for females

**DOI:** 10.1002/ece3.7815

**Published:** 2021-06-29

**Authors:** Franco Cargnelutti, Alicia Reyes Ramírez, Shara Cristancho, Iván A. Sandoval‐García, Maya Rocha‐Ortega, Lucía Calbacho‐Rosa, Freddy Palacino, Alex Córdoba‐Aguilar

**Affiliations:** ^1^ Departamento de Diversidad Biológica y Ecología Facultad de Ciencias Exactas Físicas y Naturales Universidad Nacional de Córdoba Córdoba Argentina; ^2^ Laboratorio de Biología Reproductiva y Evolución Consejo Nacional de Investigaciones Científicas y Técnicas (CONICET) Instituto de Diversidad y Ecología Animal (IDEA) Córdoba Argentina; ^3^ Departamento de Ecología Evolutiva Instituto de Ecología Universidad Nacional Autónoma de México Coyoacán México; ^4^ Grupo de Investigación en Odonatos de Colombia (GINOCO) Grupo de Investigación en Biología (GRIB) Centro de Investigación en Acarología Departamento de Biología Universidad El Bosque Bogotá Colombia

**Keywords:** copulation courtship, costs, indirect benefits, *Tenebrio molitor*

## Abstract

Postcopulatory sexual selection has shaped the ornaments used during copulatory courtship. However, we know relatively little about whether these courtship ornaments are costly to produce or whether they provide indirect benefits to females. We used the mealworm beetle, *Tenebrio molitor*, to explore this. We challenged males using an entomopathogenic fungus and compared their courtship (frequency of leg and antennal contacts to the female), copulation duration, number of eggs laid, and hatching rate against control males. Infected males copulated for longer yet they reduced their leg and antennal contacts compared to control males. However, there was no obvious relation between infection, copulation duration, and courtship with egg production and hatching success. In general, our results indicate that the ornaments used during postcopulatory courtship are condition‐dependent. Moreover, such condition dependence cannot be linked to male fitness.

## Graphical abstract

Ornaments used during postcopulatory courtship are condition‐dependent, but it is unclear whether such dependence is linked to fitness.

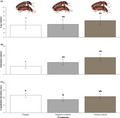

## 1 INTRODUCTION

Sexual selection is responsible for the development of highly elaborated traits, known as secondary sexual characters (SSCs) (Andersson, 1994; Cotton et al., 2006; Darwin, 1871). These SSCs are used during courtship to persuade and stimulate females to mate using visual, auditory, tactile, and/or chemical signals (Andersson, 1994; Eberhard, 1996; Mitoyen et al., 2019). Theory indicates that these SSCs are used by females as indicators of male quality (Cotton et al., 2004; Grafen, 1990; Zahavi, 1975). The fundamental idea is that the cost of producing and maintaining SSCs depends directly on the condition of males, which can be defined as their genetic potential to survive and reproduce (Kokko et al., 2002; Cotton et al., 2004; Mays Jr & Hill, 2004). While the cost of SSCs used in courtship has been well documented for traits shaped by precopulatory sexual selection (e.g., Cotton et al., 2004, 2006), this is not the case for traits shaped by postcopulatory sexual selection (i.e., behaviors related with copulatory courtship). Overall, it should be noted that there is some evidence of costs for postcopulatory traits (e.g., characteristics of the ejaculate, Cordes et al., 2015; Macartney et al., 2018; Vrech et al., 2019), but the costs of courtship traits that qualify as postcopulatory SSCs have only been proposed (Eberhard, 1996).

One conspicuous aspect of copulatory courtship that could be linked to male condition and benefits for the female is how frequently the male strokes the female using different parts of the body, such as the legs, head, and antennae (Eberhard, 1994, 1996). One example involving stroking is that of the beetle *Diabrotica undecimpunctata howardi*: Males that stroked females with their antennae more intensely during copulation were able to more effectively transfer their spermatophore compared to males that stroked less intensely (Tallamy et al., 2003). Apparently, more rapid stroking with the antennae allows the musculature around the female's sperm storage organs to distend and allow the spermatophores to pass to areas where they will be used for fertilization (Tallamy et al., 2003). However, the relationship between copulatory courtship and the fitness of males and females is not always as predicted by theory (e.g., Tallamy et al., 2003; Edvardsson & Arnqvist, 2006). While it is not a new idea that copulatory behavior may be related to some aspect of male condition (e.g., energetic competence in spiders) (e.g., Eberhard, 1996; Watson & Lighton, 1994), there are few studies that have tested it experimentally. Moreover, these studies should be accompanied by another key variable which is copulation duration. One reason is that copulatory courtship is dependent on copulation duration simply because how long a male can perform his courtship will be ultimately affected by how long the couple remains copulating. For example, an infected male spends more time in copulation if male condition affects spermatophore production (which is in turn affected by copulation duration) (e.g., Kerr et al., 2010; Reyes‐Ramírez et al., 2021).

A study system to investigate male condition, copulatory courtship (via stroking), copulation duration, cryptic female choice, and fitness consequences is the mealworm beetle, *Tenebrio molitor*. In this species, males produce pheromones before copulation to attract females (Hurd & Parry, 1991; McConnell & Judge, 2018; Rantala et al., 2003). Several results suggest that pheromones are affected by male condition (e.g., nutritional level) (Rantala et al., 2003). Nevertheless, males whose condition has been experimentally manipulated (immune challenge) were more attractive (Sadd et al., 2006; Kivleniece et al., 2010; Nielsen & Holman, 2012; Krams et al., 2014; Reyes‐Ramírez, Enríquez‐Vara, et al., 2019). Perhaps, males that increase their attractiveness in a terminal investment fashion reduce their survival (Reyes‐Ramírez, Enríquez‐Vara, et al., 2019). Interestingly, this preference for sick males led to decreased egg production and lower hatching success (Reyes‐Ramírez, Enríquez‐Vara, et al., 2019). This suggests an adjustment by females in which they invest less in the offspring of these males when they detect through signals other than pheromones that they are in poor condition (i.e., sick males) (Reyes‐Ramírez, Enríquez‐Vara, et al., 2019). Candidates for these alternative signals include male physical contact during copulation, one trait that is conspicuous and widely documented in *T. molitor*. During copulation, males rapidly tap females with their antennae and then rub the sides of the female's abdomen with the front legs (Font & Desfilis, 2003; Obata & Hidaka, 1982). If the female remains still, the male moves behind her while still rubbing the lateral margins of the female's elytra (Font & Desfilis, 2003). Once the male has achieved intromission, the male stops moving and gradually ceases the tapping with the antennae and rubbing with the front legs (Font & Desfilis, 2003; Obata & Hidaka, 1982).

In this study, we (a) manipulated male condition using fungal infection (from now on, we refer to the male infection status as male condition), allowing females to choose mates according to their condition and mate with them; (b) measured the effect of male condition on different male copulatory courtship traits (i.e., number of physical contacts with the male's legs and antennae on the female's back during copulation) and copulation duration; and (c) investigated whether these male traits influence female fitness (number of eggs laid as well as hatching success).

## 2 MATERIALS AND METHODS

### 2.1 Tenebrio molitor breeding colony

The colony was initially found by individuals from five breeding centers in the State of Mexico and Mexico City to reduce inbreeding. After 2 years, the resulting individuals were used for the experiments described below, which were maintained at an ambient temperature of 25 ± 2°C and a natural photoperiod of 12‐hr light/12‐hr darkness. The colony was maintained on a diet of wheat bran with apple slices each week as a source of water. We kept about 200 larvae in plastic containers (30.5 cm diameter × 10.5 cm height) to reduce cannibalism, as recommended by past studies (Weaver & McFarlane, 1990). Pupae were sexed based on the morphology of the eighth abdominal segment (Bhattacharya et al., 1970). Individuals were separated by sex to ensure virginity prior to the choice test.

### 2.2 Fungus cultivation and LD_50_

We used the fungus *Metarhizium robertsii* (ARSEF 2134), which was acquired from the collection of entomopathogenic fungi of the Agricultural Research Service of the United States Department of Agriculture. This entomopathogenic fungus has been shown to affect the condition of male *T. molitor* (Reyes‐Ramírez, Enríquez‐Vara, et al., 2019). Even though this fungus has been previously used in this species (e.g., Reyes‐Ramírez, Enríquez‐Vara, et al., 2019; Reyes‐Ramírez et al., 2021), we tested its viability and lethal dose as described below. Spores were transported in a solution of 10% glycerol at −80°C and were stored in Sabouraud Dextrose Agar (SDA) for later incubation for 15 days at 28°C without exposure to light. Conidiophores were carefully collected from the SDA plate and suspended in 0.03% Tween 80 solution (hereafter referred to as Tween). The suspension was mixed by vortexing for 5 min and filtered through cotton mesh to separate the conidia from the mycelium. The number of conidia and their percent viability were counted in a Neubauer chamber. We used the counting technique on the SDA plate (Goettel & Inglis, 1997), which indicated a relative viability of conidia above 95%. From the filtering, the LD_50_ (median lethal dose) was obtained, which has been previously reported by Reyes‐Ramírez, Enríquez‐Vara, et al. (2019).

### 2.3 Health treatments and application

The treatments were applied to sexually mature males (10–12 days of age; Gerber, 1976) only, 3 days before tests, and they were kept virgin until the day of the test. The following three groups were generated: (a) infected males with the entomopathogenic fungus (hereafter, fungus group), which were submerged for 5 s in a dilution of Tween 80 to 0.03% with conidia at the LC_50,_ an approximate concentration of 3 × 10^5^ conidia/ml of *M. robertsii*; (b) positive control (hereafter, Tween group), in which males were submerged for 5 s in 20 ml Tween 80 at 0.03% but with no conidia; and (c) negative control (hereafter, negative control group), males that were not manipulated during the adult stage. Finally, all animals were placed individually in 12‐well plates in an incubator at 25℃ until the choice test.

### 2.4 Choice tests

In a first step of precopulatory female choice, females chose among males of the three treatments in choice tests: (a) negative control versus. Tween; (b) fungus versus. Tween; and (c) fungus versus. negative control. We used a total of 60 different triads for each combination (60 × 3 combinations = 180 total tests using 540 different individuals). The tests were carried out in a dark room using a red‐light source, which is not detected by the insects. We used a glass Y‐olfactometer, which was adapted to connect an air pump to the end of both arms to direct the scent of each male toward the female. The olfactometer had three porous doors that allowed volatiles to be drawn down the arms toward the other end but did not allow physical contact between individuals. A female was placed at the end of the olfactometer, while each of the males of different treatments was placed in one (randomly selected) arm. The female was placed in the release port, where she was allowed to acclimatize for 2 min. At the end of that time, the door was opened to allow the female to make her choice, considering only the first side chosen by the female by reaching the end of the arm (changes of choice were not considered; (Reyes‐Ramírez, Enríquez‐Vara, et al., 2019)). The olfactometer was repeatedly cleaned with 70% ethanol to avoid the accumulation of pheromones and chemical residues. Once the choice was made, each pair was kept in isolation to prevent them from copulating until the courtship was recorded.

### 2.5 Recording of copulatory courtship

After 10 min, we placed each pair, female and the chosen male, in a glass container (12.2 cm diameter × 16.4 cm height) with a fine layer of wheat bran. During a maximum of 1 hr, the courtships that led to the first copulation were recorded. The recording was done using a 12‐megapixel camera (Samsung Galaxy S8+) with an OIS lens. Among pairs that copulated, a copulation was considered successful if it lasted for more than 30 s, since that is the approximate time that a male take to transfer a spermatophore (Gadzama & Happ, 1974). For the copulation analysis, we considered (a) the number of times the male tapped with his legs on the sides of the female's elytra; (b) the number of times the male tapped the edges of the elytra and the thorax with his antennae during copulation; and (c) copulation duration, from the moment of intromission of the aedeagus until it was removed from the female. The person who scored beetle behavior was blind to the male's treatment (infection status). If the pair fulfilled the aforementioned criteria, the female was moved to a plastic container (9 cm diameter × 7 cm tall) with 9 g of flour for 1 week, since flour is a substrate that facilitates the extraction of the eggs and is a food source for the female.

### 2.6 Effects on the progeny: number of eggs and hatching success

The females isolated after copulation were removed from the flour container after 1 week. The contents of each container were sifted to extract and count the eggs from each female. Once the eggs had been counted, the hatching rate was determined by periodically counting the number of larvae that had emerged in the days after collection (1–2 weeks after oviposition) per pair.

### 2.7 Statistical analysis

First, to test for female preferences between the two different male challenge treatments in each of the three choice tests (negative control versus. Tween, fungus versus. Tween, and fungus versus. negative control), we used a generalized linear model (GLM) with binomial error distribution. The number of males chosen from each of the three treatments was the response variable, and the treatment was the independent variable. Second, to test whether copulatory courtship and copulation duration differed depending on male experimental treatment, we used independent GLMs for the two recorded courtship behaviors (number of times the males made physical contacts with the female using their legs and antennae) and copulation duration with health treatment as the independent variable. For antenna contact behavior and leg contact behavior, we used a Poisson error distribution and log link, while for copulation duration, we used a Gaussian error distribution and identity link. Third, to determine the effect of treatment and courtship on fecundity traits, we used independent GLMs for each of the two response variables—egg number and hatching success—using Poisson error distribution (log link) and binomial error distribution (probit link), respectively. If in these models the interaction between copulatory courtship and/or copulation duration and the treatment was statistically significant, the slopes between the groups established by the treatment were compared. For this analysis, we used the emtrends function of the “emmeans” package (Lenth & Lenth, 2018). All analyses were carried out using R version 3.5.1 in RStudio 1.1.463 (Team, R.C., 2017).

## 3 RESULTS

### 3.1 Female precopulatory choice

Females did not show a preference for males of a particular treatment (*χ* ^2^ = 0.042, *p* = .97).

### 3.2 Effect of treatment on male copulatory traits

**FIGURE 1:**
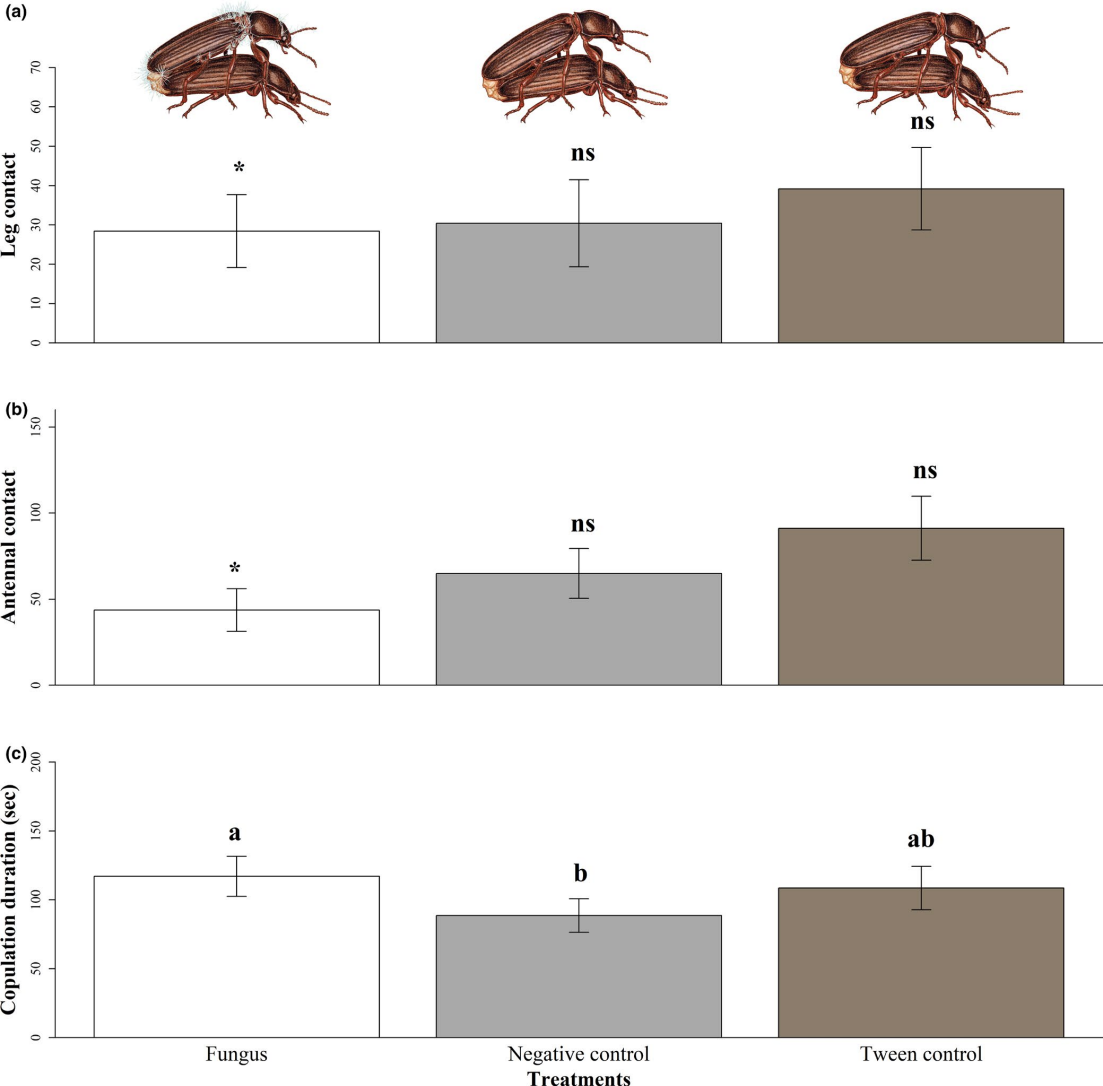
Effect of male treatment on the leg (a) and antennal (b) contacts that males performed during copulation and copulation duration (c) in *Tenebrio molitor*. Data are shown as mean ± *SE*. Asterisks indicate significant differences (a and b). Different letters indicate significant differences between treatments (c). Considering an α of 0.05

We found that all three behaviors expressed by males differed according to their treatment: leg contact (*χ* ^2^ = 85.09, *p* < .001; Figure 1a), antennal contact (*χ* ^2^ = 765, *p* < .001; Figure 1b), and copulation duration (*F* = 3.56, *p* = .01; Figure 1a). Fungus‐infected males had fewer antennal contacts than negative control males (*z* = −22.35, *p* < .001) and Tween males (*z* = −46.36, *p* < .001), and Tween males had more antennal contacts than negative control males (*z* = 22.39, *p* < .001). Likewise, fungus‐infected males had fewer leg contacts than negative control males (*z* = −2.84, *p* =.01) and Tween males (*z* = −15.06, *p* < .001). Tween males had more antennal contacts than negative control males (*z* = 11.23, *p* < .001). Fungus‐infected males copulated for longer than negative control males (*t* = 28.43, *p* = .02) but did not differ from Tween males (*t* = 8.48, *p* = .68). Copulation duration was similar between negative control and Tween males (*z* = 19.94 *p* = .16).

### 3.3 Effect of treatment and copulatory traits on egg number

We found a significant relationship between leg contact behavior (*χ* ^2^ = 6.98, *df* = 2, *p* = .030), antennal contact behavior (*χ* ^2^ = 25.25, *df* = 2, *p* < .001), and copulation duration (*χ* ^2^ = 38.48, *df* = 2, *p* < .001) with the number of eggs laid by the females considering the experimental treatment (i.e., we found a significant interaction between the copulatory courtship behaviors and copulation duration and the experimental treatment). Individuals of the fungus treatment and Tween control treatment ended up giving rise to fewer eggs. As individuals of the negative control treatment increased copulation duration, their female partners laid more eggs. However, although both treatments have the same slope (i.e., negative slope), a steeper slope can be seen (i.e., a faster drop in the number of eggs laid with increasing copulation duration) in the Tween control treatment. Also, we found that an increase in leg and antennal contacts made by males from the fungus treatment and negative control treatment leads to a decrease in egg number, while the opposite effect was found for Tween control males (Table 1).

**TABLE 1:** Table of contrasts between slopes resulting from the interaction between treatment (i.e., negative control, fungus, Tween) and copulatory courtship (i.e., number of leg contacts, number of antennal contacts) and copulation duration (response variable = number of eggs laid)

Contrast	Estimate	*SE*	*z*	*p*
**Treatments × leg contacts**				
Fungus–negative	−0.00403	0.00264	−1.526	.2786
Fungus–tween	−0.00670	0.00261	−2.570	.**0274**
Negative–tween	−0.00268	0.00220	−1.216	.4438
**Treatments × antennal contacts**				
Fungus–negative	−0.00313	0.00194	−1.614	.2395
Fungus–tween	−0.00769	0.00176	−4.383	**<.0001**
Negative–tween	−0.00457	0.00143	−3.197	.**0040**
**Treatments × copulation duration**				
Fungus–negative	−0.00777	0.00176	−4.421	**<.0001**
Fungus–tween	0.00338	0.00192	1.755	.1850
Negative–tween	0.01115	0.00185	6.013	**<.0001**

Note

*p*‐values in bold indicate significant differences between slopes with an α of .05.

### 3.4 Effect of treatment and copulatory traits and copulation duration on egg hatching success

We found a significant relationship between leg contact behavior (*χ* ^2^ = 9.83, *df* = 2, *p* = .007) and copulation duration (*χ* ^2^ = 9.57, *df* = 2, *p* = .008) with hatching success considering the experimental treatment (as mentioned above, this means that we found a significant interaction between copulatory courtship behaviors and copulation duration and experimental treatment). However, we did not find an effect of the antennal contact behavior on hatching success (*χ* ^2^ = 2.169, *df* = 1, *p* = .141). As individuals of the negative control treatment increased copulation duration, they gained in hatching success, while those of the fungus treatment and Tween control treatment decreased their hatching success. However, although both treatments have the same slope (i.e., negative), individuals of the fungus treatment had an initially lower proportion of egg hatching than the Tween control treatment (i.e., a lower intercept). Finally, as males of the negative control treatment and the fungus treatment increased leg contacts, their hatching success decreased. Although both treatments have the same slope (i.e., negative), individuals of the fungus treatment have an initially lower hatching success than the negative control treatment (i.e., a lower intercept), while those of the Tween control treatment did not show such an effect (Table 2).

**TABLE 2:** Table of contrasts between slopes resulting from the interaction between treatment (i.e., negative control, fungus, Tween) and copulatory courtship (i.e., number of leg contacts, number of antennal contacts) and copulation duration (response variable = proportion of egg hatching)

Contrast	Estimate	*SE*	*z*	*p*
**Treatments × leg contacts**				
Fungus–negative	−0.01327	0.00440	−3.017	**0.0072**
Fungus–tween	−0.00966	0.00416	−2.324	0.0525
Fungus–tween	0.00361	0.00290	1.246	0.4261
**Treatments × copulation duration**				
Fungus–negative	0.000336	0.00261	0.129	0.9909
Fungus–tween	0.008321	0.00299	2.784	**0.0148**
Negative–tween	0.007985	0.00298	2.677	**0.0203**

Note

*p*‐values in bold indicate significant differences between slopes with an α of .05.

## 4 DISCUSSION

Females of *T. molitor* did not show preferences for males of different conditions. For whatever reason, our treatment did not affect precopulatory preferences, such that females were not able to distinguish males according to their condition. In this situation, it is possible that infected males expressed volatile pheromones with the same intensity and/or quality as healthy males. Our results differ from other studies where *T. molitor* females prefer to copulate with males in poor condition (Kivleniece et al., 2010; Krams et al., 2014; Nielsen & Holman, 2012; Reyes‐Ramírez, Rocha‐Ortega, et al., 2019; Sadd et al., 2006). The interpretation of those studies was that males infected with the fungus engaged in terminal investment, emitting more pheromones at the cost of reduced survival (e.g., Reyes‐Ramírez, Reyes‐Ramírez, et al., 2019; Reyes‐Ramírez, Rocha‐Ortega, et al., 2019). As for our current results, only few studies have found that females may not use odor cues to determine the condition of males (Newman & Buesching, 2019; Wyatt, 2017). In other respects, infected males copulate for longer than healthy males. Nevertheless, they do not differ from Tween males in terms of copulation duration. Also, copulation duration was similar between negative control and Tween males. The three most commonly described mechanisms as for why males would increase copulation duration are that males (a) transfer more sperm (e.g., Anderson & Hebets, 2017); (b) have more time to stimulate the female (e.g., Andrés & Rivera, 2000), and/or (c) reduce females' remating opportunities (Wulff & Lehmann, 2016). In the case of our study species, these explanations are not totally applicable because the copulatory mechanisms are unknown, so we will speculate to understand the effects of the fungus during copulation. One explanation is that how long a couple remains together depends on the production of the spermatophore. In this sense, it is possible that infected males require more time for spermatophore transfer given their deteriorated health (e.g., Hughes et al., 2000; Vahed et al., 2011; Duplouy et al., 2018). A second explanation is that sick males were not able to stimulate females, which could lead to males spending more time copulating but without being able to make contacts to and, thus, provide enough stimulation to the female (Eberhard, 1996). This seems to be the case since infected (and Tween) males produced fewer leg and antenna courtship contacts than negative control males. Related to this, stroking the female acts as a copulatory courtship in a variety of insects (Eberhard, 1996; Edvardsson & Göran, 2000) eliciting various female physiological and/or behavioral responses in order to favor male paternity (for a detailed review of the more than 20 possible mechanisms in females, see Eberhard, 1996). However, it is possible that the cost of infection cannot be observed through the duration of copulation but rather through the number or quality of sperm transferred or even by the quality of the ejaculate (e.g., Kerr et al., 2010). Considering the terminal investment hypothesis, infected males may invest in larger or better‐quality ejaculates, as observed in the same study species (Reyes‐Ramírez et al., 2021). The simplest mechanism to explain a link between copulatory courtship and the above phenomena is that producing the leg and antenna movements is energetically demanding, a cost that sick males cannot afford. Actually, pre‐ and postcopulatory courtship can be energetically costly in arthropods. This is the case of, for example, the intromission of genitals by the male (genital courtship) and abdominal vibrations (nongenital courtship) in spiders (Cargnelutti, 2020; Watson & Lighton, 1994) and precopulatory courtship behaviors in insects (e.g., Wedell, 2010; Mowles & Jepson, 2015). Thus, the fact that infected males spent more time in copulating may be a way of compensating for their decreased leg and antennal performance.

In terms of the link between copulatory courtship traits and copulation duration and reproductive success in *T. molitor*, the scenario is complex. Healthy males that copulated for longer ended up fathering more eggs and having a higher hatching success although infected males copulated for longer than healthy males (see above). However, when infected and healthy males prolonged the copulation duration, their partners produced fewer eggs and lower hatching success. This opens the question of whether females assess some other male aspects and not only copulation duration. Although one would expect one such aspect to be related to copulatory courtship traits, we did not find a difference in the outcome of copulatory courtship between healthy and sick males. It may simply be that the aspects we measured did not include those that are being selected. Surprisingly, an increase in leg and antennal contacts led to an increase in egg number in Tween males but the opposite for the other two treatments. Other studies in the group have shown striking results, beyond what was expected when using Tween as a control group (e.g., Reyes‐Ramírez, Reyes‐Ramírez, et al., 2019; Reyes‐Ramírez, Rocha‐Ortega, et al., 2019). Therefore, we cannot rule out that Tween may have some effect on individuals. However, it is difficult to understand why females would be responding favorably to male's copulatory courtship belonging to this treatment in comparison with healthy males. Generally speaking, a positive effect was suggested between the copulatory courtship performed by the males and their reproductive success (Eberhard, 1996; Edvardsson & Göran, 2000; Sirot et al., 2007; Barbosa, 2009). However, some studies have not found an effect between these two parameters (Tallamy et al., 2003; Edvardsson & Arnqvist, 2006; Fedina & Lewis, 2015; Eberhard et al., 2020).

Although fungus‐treated males performed a less intense copulatory courtship, the infection did not appear to affect reproductive success. Considering that such copulatory behaviors could be energetically costly (for more details of costly behaviors, see Byers et al., 2010), one may wonder why males still perform such courtship. Furthermore, our results also indicate that rather than favor courtship intensity, females seem to penalize males who court more. To our knowledge, there are no studies that have shown this. However, it is possible that females penalize males that exceed a threshold. There is support for such threshold‐related female sexual responses in spiders (Peretti et al., 2006). In this regard, *T. molitor* females are not passive and have been observed to walk during copulation (all authors' unpub. observations). Whether male and female copulatory behavior can be used as a way of copulatory dialogue in *T. molitor* awaits further investigation.

To conclude, our study indicates that SSCs in the form of postcopulatory traits are costly to produce, which matches what we know of SSCs at the precopulatory level (reviewed, e.g., in Hill, 2015). However, such costs of maintaining copulatory courtship do not necessarily translate into males' reproductive success. It may be that we missed to measure some other traits that are being selected during copulation. Some traits may be related to the volatiles that males produce at the level of pheromones or cuticular hydrocarbons. In any case, the question is still open of whether the condition‐dependent nature of postcopulatory SSCs is linked to male fitness.

## CONFLICT OF INTEREST

None declared.

## AUTHOR CONTRIBUTIONS

**Franco Cargnelutti:** Conceptualization (equal); Data curation (equal); Formal analysis (equal); Investigation (equal); Methodology (equal); Writing‐original draft (equal); Writing‐review & editing (equal). **Alicia Reyes‐Ramírez:** Conceptualization (equal); Investigation (equal); Methodology (equal); Resources (equal); Writing‐original draft (equal). **Shara Cristancho:** Data curation (equal); Formal analysis (equal); Investigation (equal); Methodology (equal). **Ivan Sandoval:** Investigation (equal); Methodology (equal); Resources (equal). **Maya Rocha:** Data curation (equal); Formal analysis (equal); Writing‐review & editing (equal). **Lucía Calbacho‐Rosa:** Conceptualization (equal); Investigation (equal); Writing‐original draft (equal); Writing‐review & editing (equal). **Fredy Palacino:** Conceptualization (equal); Data curation (equal); Writing‐original draft (equal). **Alex Córdoba‐Aguilar:** Conceptualization (lead); Funding acquisition (lead); Investigation (lead); Methodology (lead); Project administration (lead); Resources (lead); Supervision (lead); Validation (equal); Visualization (equal); Writing‐original draft (lead); Writing‐review & editing (lead).

### DATA AVAILABILITY STATEMENT

Data can be accessed from 10.6084/m9.figshare.14712954.
